# Minimally Invasive Resection of an S3 Osteoid Osteoma Using an Intraoperative O-Arm: A Technical Note

**DOI:** 10.7759/cureus.18262

**Published:** 2021-09-25

**Authors:** Alejandro Matus, Charles Touchette, Tarek Sunna, Daniel Shedid, Shadi Bsat, Hani Chanbour, Alexander G Weil

**Affiliations:** 1 Neurological Surgery, Herbert Wertheim College of Medicine, Florida International University, Miami, USA; 2 Neurosurgery, Sherbrooke University, Quebec, CAN; 3 Neurosurgery, American University of Beirut Medical Center, Beirut, LBN; 4 Neurosurgery, Centre hospitalier de l'Université de Montréal, Montreal, CAN; 5 Faculty of Medical Sciences, Lebanese University, Beirut, LBN; 6 Department of Surgery, Division of Neurosurgery, Sainte-Justine University Hospital Center, Montreal, CAN

**Keywords:** vertebral tumor, sacrum, o-arm, minimally invasive surgery, osteoid osteoma

## Abstract

Osteoid osteomas are benign primary bone tumors that typically arise in posterior vertebrae of the spine. For patients with severe pain or those poorly controlled with non-steroidal anti-inflammatory drugs, surgical management is the mainstay of treatment. The recommended surgical treatment option is complete open excision, although minimally invasive CT-guided percutaneous excision and CT-guided radiofrequency ablation have been reported. Open resection can result in prolonged hospital stays, activity restrictions, and possible spinal destabilization. We sought to utilize a lateral minimally invasive approach. We highlight the importance of aggressive surgical resection and the utility of using fluoroscopy and O-arm guidance to optimize the extent of resection. We report a pediatric case of a 12-year-old male who presented with an S3 osteoid osteoma. The patient underwent a minimally invasive resection with complete resection and confirmation of the histopathologic diagnosis. Postoperative imaging showed complete resection of the tumor. The patient went home five hours after surgery with return to daily activities; his symptoms resolved completely. However, the patient had symptomatic recurrence and underwent a second more aggressive minimally invasive resection using O-arm guidance. At the current three-month follow-up, the patient is symptom- and tumor-free. The minimally invasive resection of a pediatric sacral osteoid osteoma is a valid alternative to standard open resection and is associated with a decreased blood loss, decreased length of stay in the hospital, and decreased time to full functional recovery. The pitfalls are learning curve and risk of incomplete resection that can be counterbalanced with an intraoperative O-arm to guide resection and confirm complete excision.

## Introduction

Osteoid osteomas are benign osteoblastic tumors characterized by the formation of mature bone by the tumor cells [1]. They are found in the spine in less than a quarter of cases [2]. Surgery is indicated when patients desire definitive treatment or when conservative treatment failed to control the pain [1]. Minimal invasive surgery (MIS) has proved to be a valid alternative to many conventional open approaches to the spine for multiple types of surgical approaches, including lumbar osteoid osteomas [3].

We report the case of a 12-year-old boy presenting with an S3 osteoid osteoma. His tumor was located medially in the posterior aspect of the S3 vertebral body, adjacent to the spinal canal. Neither radiofrequency ablation nor open en bloc was considered safe options. A minimally invasive resection was performed and allowed for complete resection, and confirmation of the histopathological diagnosis. Although the postoperative scan confirmed complete resection of the lesion, the patient experienced recurrence, and underwent a subsequent resection using O-arm guidance. The patient is now asymptomatic and disease-free. We highlight the importance of aggressive surgical resection when periosteal reaction is present and the importance of an O-arm to achieve complete resection when carrying out MIS resection.

## Case presentation

**Figure 1:**
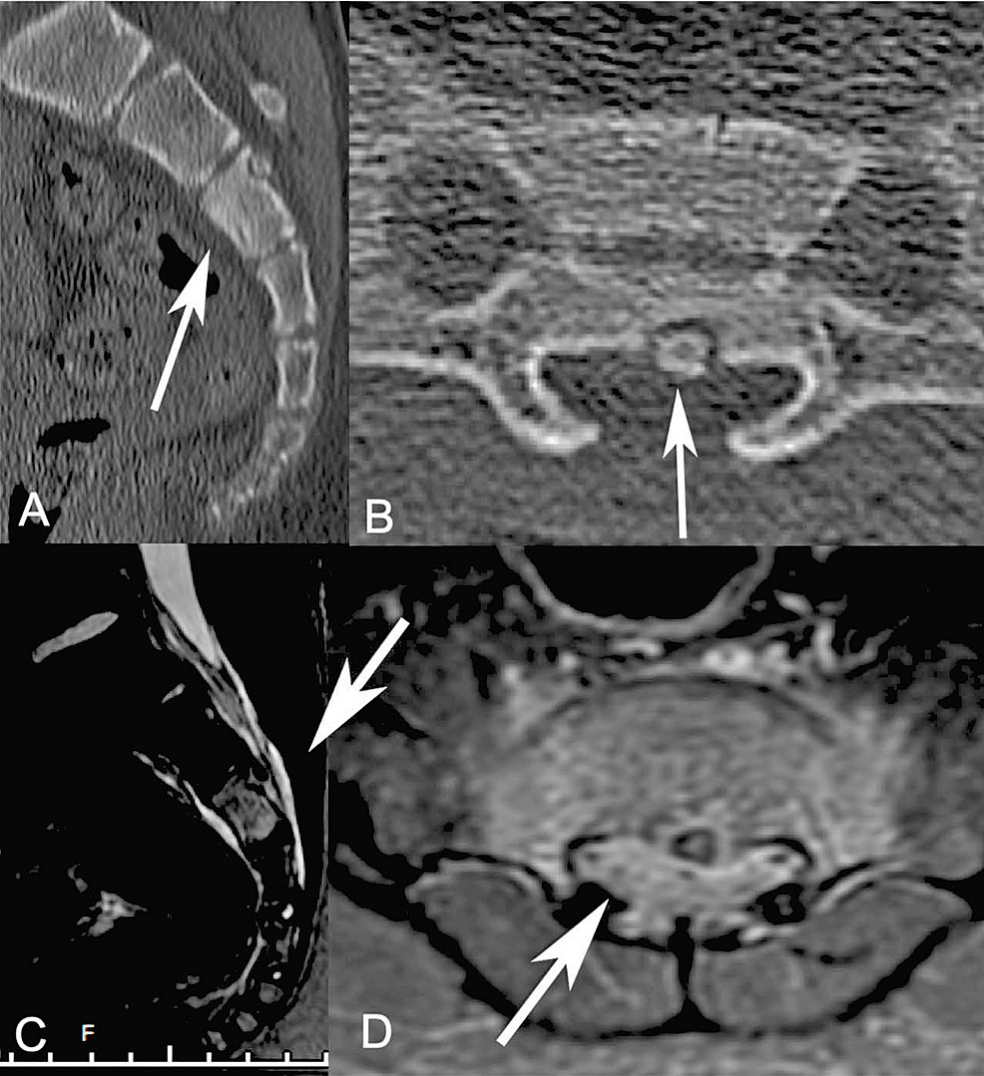
Pre-operative imaging: lumbo-sacral CT scan (A, B) and MRI (C, D) showing an osseous lesion originating from the posterior S3 body

A 12-year-old boy, previously healthy, presented with a two-month history of progressive, severe lower back pain worse at night. His physical examination revealed pain on palpation of the sacral region. A plain radiograph of the spine showed a small S3 osteolytic lesion with reactive sclerosis. A CT scan showed a sclerotic lesion medially in the posterior portion of the S3 vertebral body with compression of sacral nerve roots (Figures 1A, 1B). Magnetic resonance imaging (MRI) showed the nidus at the medial posterior portion of S3 associated with bone marrow edema (Figures 1C, 1D). The bone scan confirmed a round focus of hypermetabolism at S3. Due to the size of the lesion and radiological characteristics, a presumptive diagnosis of osteoid osteoma was given. Minimally invasive biopsy and resection was proposed to confirm the diagnosis and perform a complete resection while avoiding sacrectomy, fusion, and sacral nerve root damage.

**Figure 2:**
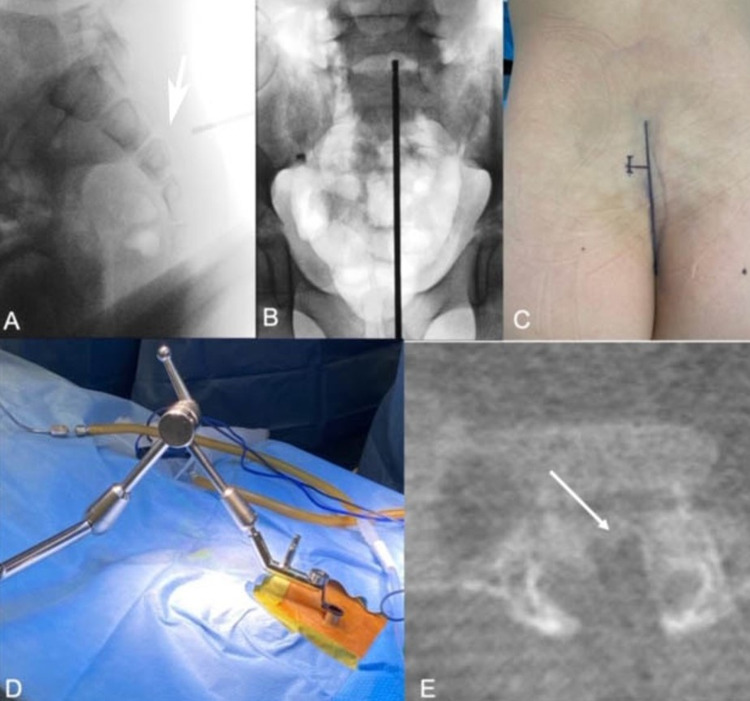
Image showing intraoperative fluoroscopy to localise the S3 level (A) and midline (B) and the final planned 2-cm medial incision (C); placement of dilatation tubes (D); Post-resection CT showing the area of resection (arrow) (E)

In prone position, fluoroscopy was used to identify the midline and the S3 level (Figures 2A, 2B). A longitudinal incision was performed at midline, and 1.5-2 cm above the S3 level (Figure 2C). The muscular aponeurosis was incised parallel and slightly medially to the skin incision. A series of dilators were introduced to split the paraspinal muscles, until an 18-mm-wide METRx expandable tubular retractor (Medtronic Sofamor Danek, Memphis, TN) could be placed at the junction of the median sacral crest and medial sacral crest of S3 (Figure 2D). Adequate positioning was confirmed with intraoperative CT fluoroscopy of the O-arm (Figure 2E). Under microscopic visualization, the S3 space and sacroiliac joints were localized. The pedicle of S3 was identified, penetrated, and resected. A sacral laminectomy was performed, exposing the thecal sac. A suction-retractor was used to retract the dura and expose the foramen along with the tumor in the superior portion of the surgical field. Intralesional piecemeal resection of the tumor was performed. Specimens were sent to pathology. Intraoperative CT fluoroscopy confirmed resection into the extra-foraminal space. Intraosseous and epidural methylprednisolone was injected. Standard surgical closure was performed.

**Figure 3:**
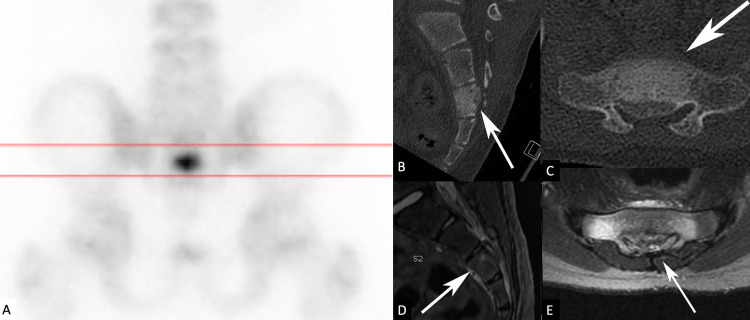
Bone scan (A) showing increase captation in the sacral region. CT scan (B, C) and MRI (D, E) showing recurrence of the bony lesion with periosteal reaction (arrows)

**Figure 4:**
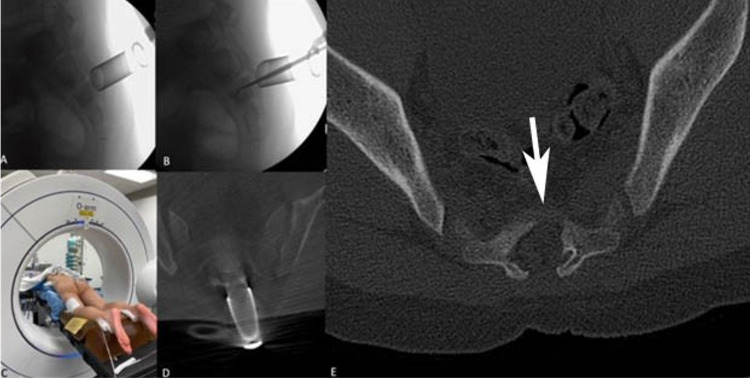
Repeat surgery showing positioning intraoperative lateral fluoroscopy to confirm final position of the retractor tube (A) and inspection of the extent of resection. Image showing O-arm placement (C) and confirming position (D); the postoperative CT scan shows extensive and complete resection (E)

The postoperative course was uneventful and a complete symptom resolution was achieved. The patient was discharged home five hours after surgery, and quickly returned to his daily activities. Pathological analysis confirmed the initial diagnosis of osteoid osteoma. A complete resection was confirmed on the postoperative CT. However, at six-month follow-up, the patient had recurrence of low back pain. Repeat imaging with bone scan (Figure 3A), CT (Figures 3B, 3C), and MRI (Figures 3D, 3E) showed a recurrence of the osteoma with periosteal reaction. The patient underwent an outpatient MIS resection of the tumor under fluoroscopy and O-arm guidance (Figures 4A-4D). The intraoperative O-arm confirmed the complete resection of the lesion.

## Discussion

Osteoid osteoma of the sacrum is a very rare occurrence. Traditionally, open resection by a posterior approach is used for the surgical treatment of spinal osteoid osteoma. However, open resection of spinal osteoid osteoma can be challenging, and typically requires significant healthy tissue resection and wide surgical resections of the bony structure to ensure removal of the nidus. This can increase morbidity for the patient in the forms of significant blood loss, postoperative pain, increased hospital stays, and perhaps even necessitating fusion to restore spinal stability [4]. Our case involved the posterior aspect of the S3 vertebral body, which additionally made traditional open surgical resection too hazardous to proceed with due to potential difficulties with neurological damage, identifying the nidus, increased risk for postoperative infections and wound dehiscence, as well as spinal instability. To avoid the complications of a posterior or anterior open approach, an alternative treatment option for our patient was necessary.

As of our case, the MIS technique was especially helpful in locating and removing the nidus safely. This technique allowed for the minimal dissection of the surrounding tissue; neuromonitoring was used to ensure the preservation of all nearby nervous structures. Intraoperative fluoroscopy combined with the expandable tube led to the great exposure of the lesion and greater confidence in complete resection. On the other hand, MIS offered a limited visual field, which increased the risk of incomplete resection of the tumor. This led to an additional procedure.

Fluoroscopy has revolutionized spinal surgery instrumentations and increased accuracy. The C-arm was first used to render 2D images, and then O-arm fluoroscopy was introduced with 3D reconstruction of the surgical field. The latter has reduced operative time and minimized the surgeon’s exposure to radiation [5,6].

MIS techniques for tumors of the spine have sought to decrease paraspinal tissue damage while still treating the underlying pathology. Additionally, Regev et al. showed similar positive results with minimally invasive open resection of 14 benign osseous tumors, including five osteoid osteomas [4]. A recent study demonstrated the feasibility of MIS resection in the treatment of lumbar osteoid osteoma [3]. Image-guided percutaneous radiofrequency ablation (RFA), a type of minimally invasive technique, uses high-frequency alternating current through an RF probe directed to the patient [6]. RFA was deemed unsafe due to the location of the nidus in the posterior aspect of the sacrum, which carried too high of a risk of heat-induced neurovascular damage to nearby nerve roots and arteries.

While there are limitations to MIS, the risks and safety concerns of open sacral resection and RFA as outlined earlier made minimally open excision the only suitable technique to avoid major complications and functional impairment, as well as to offer a quick recovery. MIS with an O-arm should be considered as a valuable alternative for the treatment of osteoid osteoma and other benign osseous tumors of the spine.

## Conclusions

Osteoid osteoma of the sacrum adjacent to the spinal canal presents a technical challenge for resection that precludes the use of RFA or traditional en bloc resection due to safety concerns. Our case shows the utility of MIS techniques in a successful complete resection of a sacral osteoid osteoma while simultaneously preserving bladder/bowel function and lumbopelvic stability, and also decreasing blood loss and hospital stay compared to traditional methods. Minimally invasive techniques with an O-arm should be considered as a valuable alternative for the treatment of osteoid osteomas and other benign osseous tumors of the spine.
